# Herpes simplex virus colitis mimicking acute severe ulcerative colitis: a case report and review of the literature

**DOI:** 10.1093/jscr/rjad225

**Published:** 2023-04-27

**Authors:** Marionna Cathomas, Robert Rosenberg, Emanuel Burri, Marianna Javier-Gonzalez, Achim Weber, Magdalena Filipowicz Sinnreich, Gieri Cathomas, Raffaele Galli

**Affiliations:** Department of Surgery, Cantonal Hospital Baselland, Liestal, Switzerland; Department of Surgery, Cantonal Hospital Baselland, Liestal, Switzerland; Department of Gastroenterology and Hepatology, Medical University Clinic, Cantonal Hospital Baselland, Liestal, Switzerland; Department of Internal Medicine, Cantonal Hospital Baselland, Liestal, Switzerland; Department of Pathology and Molecular Pathology, Institute of Molecular Cancer Research, University Hospital Zurich, Zurich, Switzerland; Department of Gastroenterology and Hepatology, Medical University Clinic, Cantonal Hospital Baselland, Liestal, Switzerland; Department of Pathology, Cantonal Hospital Baselland, Liestal, Switzerland; Institute of Tissue Medicine and Pathology, University Bern, Bern, Switzerland; Department of Surgery, Cantonal Hospital Baselland, Liestal, Switzerland

**Keywords:** Ulcerative colitis, Herpes simplex virus, Herpes colitis, Infection in immunocompromised patients

## Abstract

A 60-year-old female patient with longstanding left-sided ulcerative colitis presented with symptoms mimicking an acute flare and developed a colonic perforation shortly after starting steroid treatment. Following left hemicolectomy and Hartmann’s procedure, rescue treatment with infliximab was started. Within a few days, the patient developed hepatic failure. Histology and immunohistochemistry of the specimen revealed extensive necrotizing herpes simplex virus colitis, and liver biopsy demonstrated herpes simplex virus hepatitis. Sixteen days after admission, the patient died from multiorgan failure. This compelling case of severe herpes simplex virus colitis raises awareness of a rare but potentially detrimental infection in patients with inflammatory bowel disease.

## INTRODUCTION

The clinical course of inflammatory bowel disease (IBD) can be complicated by viral reactivation or superinfection that must be excluded during clinical workup [1]. The most frequent viral infection is cytomegalovirus (CMV), with a reported prevalence of up to 34% [2]. In contrast, other herpes viruses, including herpes simplex virus (HSV), are rare, unexpected causes of deterioration in IBD patients [3–9].

We present the case of a patient with left-sided ulcerative colitis (UC) operated for colonic perforation secondary to HSV colitis, who developed a systemic HSV infection while on treatment with steroids and infliximab.

## CASE REPORT

A 60-year-old female patient with a 20-year history of left-sided UC presented to the emergency department (ED) with lower abdominal pain and bloody diarrhea up to 10 times daily. For most of her long-standing disease, she had experienced intermittent mild symptoms, treated solely with alternative medicine. Three days before presentation to the ED, the patient returned from a 2-week vacation in an Eastern African country. During this stay, diarrhea worsened, and she started taking budesonide rectal foam and over-the-counter ciprofloxacin.

Upon presentation to the ED, physical examination revealed lower abdominal tenderness without signs of peritonitis. Laboratory tests showed a C-reactive protein (CRP) of 166 mg/l. Since the patient refused admission, outpatient treatment with oral prednisone 60 mg/day in combination with oral and rectal 5-amino-salicylic acid (5-ASA) was prescribed. The patient returned to the ED 2 days later due to persistent symptoms and was hospitalized. Multiplex polymerase chain reaction (PCR) testing of stool samples showed no microbial infection.

**Figure 1:**
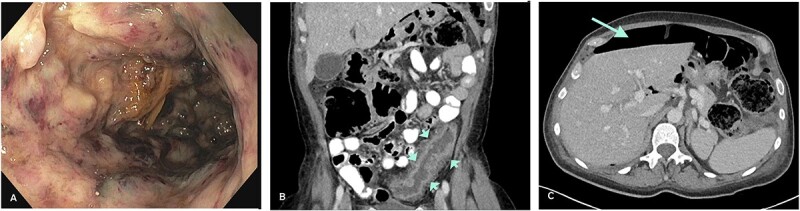
(**A**) Severe colitis with deep ulcerations on endoscopy. (**B**) Left-sided colonic wall thickening (CT-scan; short arrows). (**C**) Intraperitoneal air indicative of hollow viscus perforation (CT-scan; long arrow).

**Figure 2:**
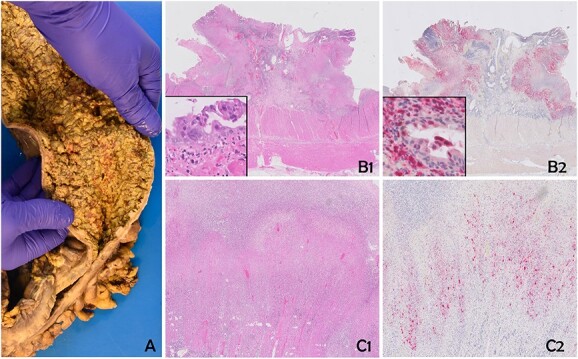
The specimen shows severe diffuse necrotizing colitis (**A**). Histology reveals extensive ulcerous colitis with deep-reaching ulcers and mural necrosis (**B1** and **C1**). Multinucleated epithelial cells with eosinophilic inclusions can be discerned (insert B1). Immunohistochemistry for human HSV confirms a widespread infection of the mucosa and the mural part of the colonic wall (**B2** and **C2**). Epithelial cells, as well as stromal cells, are positive (insert B2). B1 and C1: Hematoxylin and Eosin; C1 and C2: Immunohistochemistry for HSV (Chromogen: Fast Red).

A sigmoidoscopy revealed severe inflammation with deep ulcers and patches of mucosal necrosis (Fig. 1A). Biopsies from the rectum and distal colon showed severe inflammation and necrosis, and immunohistochemistry (IHC) for CMV was negative. Treatment was changed to intravenous methylprednisolone 50 mg/day and metronidazole/ciprofloxacin. An abdominal computed tomography (CT) scan revealed a left-sided colonic wall thickening with free intra-abdominal air (Fig. 1B and C). Emergency laparoscopy showed purulent peritonitis with a perforation of the descending colon. Due to the lack of inflammation of the right colon and according to the patient’s preference to avoid subtotal colectomy, a laparoscopic left hemicolectomy with formation of an end colostomy (Hartmann’s procedure) was performed. Postoperatively, the patient’s condition improved rapidly. Biologic therapy with infliximab was started 2 days after surgery to address the severe proctitis and potential inflammation in the remnant colon. Gross specimen examination showed a severe diffuse necrotizing colitis, and histology revealed an extensive ulcerating colitis with pseudomembranous character with patchy, transmural necrosis and perforation in the proximal descending colon (Fig. 2A, B1 and C1). The mucosal inflammation ended abruptly adjacent to the perforation, and the oral resection margin showed normal mucosa, whereas the aboral margin was heavily inflamed and partially necrotic.

On the third postoperative day, liver enzymes increased markedly. A CT scan of the abdomen showed hypodense liver lesions, possibly due to hepatic necrosis. Within 24 hours, liver function tests further deteriorated, and the patient became encephalopathic. She was transferred to a transplant center for evaluation of urgent liver transplantation.

In the further workup, serologic tests were positive for HSV-1 + 2 immunoglobulin G and M, and a liver biopsy showed HSV hepatitis. HSV viremia with 60 million copies/ml was detected, suggesting a systemic herpes virus infection. Clinical examination revealed erosive genital lesions positive for HSV-2. IHC of the colonic specimen was performed and confirmed HSV infection in the ulcers and necrotic areas (Fig. 2B2 and C2). Further molecular analysis of the colonic tissue by type-specific PCR revealed HSV-2.

Despite antiviral treatment and extensive critical-care management, the patient died of multiple organ failure 16 days after the initial presentation.

## DISCUSSION

Opportunistic infections are a significant safety concern in patients with IBD. A well-recognized complication is CMV colitis, which is associated with significant morbidity and poor outcome [10]. However, the role of other herpesviruses in patients with IBD is less clear, and overt HSV colitis is exceedingly rare.

The prevalence in the general population of HSV-1 and HSV-2 is up to 98% and 20%, respectively [11]. In immunocompetent patients, HSV infections occur as self-limiting localized lesions [12]. In contrast, immunocompromised patients may develop severe localized or systemic HSV infections with significant morbidity and mortality [13].

HSV-2 proctitis is common in men having sex with men; however, in immunocompetent individuals, the infection rarely extends above 15 cm of the anal verge [14]. In the described case, an HSV-2-positive vulvar lesion indicates a potentially primary florid HSV infection at the time of clinical exacerbation. The patient never had conventional immunosuppression and only took rectal budesonide and high-dose oral prednisone 4 days before surgery. Considering the extensive HSV colitis at surgery, it is likely that the HSV colitis was already present at the first visit and aggravated in the following days after starting systemic corticosteroids.

Despite the high seroprevalence of HSV worldwide, HSV colitis is a rarity. We identified only seven reports of HSV colitis in patients with IBD in the English literature [3–9]. All patients presented with symptoms suggestive of acute severe UC, and initial treatment consisted of corticosteroids followed by cyclosporine [4], anti-tumor necrosis factor-inhibitors [7], or combination therapy with tacrolimus and azathioprine [3]. The diagnosis of HSV colitis was made on biopsy in only one case [6] and similar to our case, bowel perforation with subsequent surgery occurred in three patients and HSV was therefore detected with delay [5–7]. In three cases, HSV-2 was found [6, 8, 9]; in the remaining patients, the genotype was not characterized [3–5, 7].

In summary, although severe HSV colitis is a rare complication in patients with IBD, physicians treating IBD patients should be aware of the potential risk of severe HSV colitis following immunosuppressive therapy. In line with the most recent ECCO guidelines of 2021, all patients should be asked for recurrent or florid HSV infection before starting immunosuppressive treatment. In case of positive history, antiviral prophylaxis should be considered [15]. A thorough medical history and clinical examination can lead to rapid diagnosis and early initiation of antiviral treatment.

## Data Availability

The data underlying this article cannot be shared publicly due to privacy reasons of the individual in the present short report. The data will be shared on reasonable request to the corresponding author.
